# Discharging the complex patient - changing our focus to patients’ networks of care providers

**DOI:** 10.1186/s12913-021-06841-2

**Published:** 2021-09-10

**Authors:** Laurent Perrault-Sequeira, Jacqueline Torti, Andrew Appleton, Maria Mathews, Mark Goldszmidt

**Affiliations:** 1 Schulich School of Medicine & Dentistry, Western University, London, ON Canada; 2 Centre for Education Research & Innovation – Western University, London, ON Canada

**Keywords:** Qualitative research, Patient-centred care, Patient safety, Discharge planning, Hospital medicine, Primary care

## Abstract

**Background:**

A disconnect exists between the idealized model of every patient having a family physician (FP) who acts as the central hub for care, and the reality of health care where patients must navigate a network of different providers. This disconnect is particularly evident when hospitalized multimorbid patients transition back into the community. These discharges are identified as high-risk due to lapses in care continuity. The aim of this study was to identify and explore the networks of care providers in a sample of hospitalized, complex patients, and better understand the nature of their attachments to these providers as a means of discovering novel approaches for improving discharge planning.

**Methods:**

This was a constructivist grounded theory study. Data included interviews from 30 patients admitted to an inpatient internal medicine service of a midsized academic hospital in Ontario, Canada. Analysis and data collection proceeded iteratively with sampling progressing from purposive to theoretical.

**Results:**

We identified network of care configurations commonly found in patients with multiple medical comorbidities receiving care from multiple different providers admitted to an internal medicine service. FPs and specialists form the network’s scaffold. The involvement of physicians in the network dictated not only how patients experienced transitions in care but the degree of reliance on social supports and personal capacities. The ideal for the multimorbid patient is an optimally involved FP that remains at the centre, even when patients require more subspecialized care. However, in cases where a rostered FP is non-existent or inadequate, increased involvement and advocacy from specialists is crucial.

**Conclusions:**

Our results have implications for transition planning in hospitalized complex patients. Recognizing salient network features can help identify patients who would benefit from enhanced discharge support.

**Supplementary Information:**

The online version contains supplementary material available at 10.1186/s12913-021-06841-2.

## Background

In the Canadian, decentralized, universal, publicly funded health system, there is a disconnect between the idealized model of every patient having a Family Physician (FP)^1^ acting as the central hub for care and the reality where many patients receive care from a network of providers, in which an FP may only play a minor role [1–3]. This disconnect is particularly salient when patients admitted to hospital – especially those with multimorbidity who may be supported by multiple clinicians [4] – are discharged back into the community. These are high-risk events due to their potential for medical error [5]. Initiatives developed for enhancing safety and reducing error in this context typically focus on a combination of strategies including: patient education; [6] communication with the receiving FP; [7] predicting high-risk readmission patients[8] and; post-discharge clinics [9]. While there has been some improvement in outcomes as a result, in many contexts, quality and safety concerns persist and readmission rates remain high [10]. To date, the quality and configuration of a patient’s network of providers have largely been ignored. Exploring network configuration appears to be a viable path for identifying a novel approach to improving discharge planning and, ultimately, improving quality and safety.

While the idea of varying networks of care is not new, there is a dearth of research exploring their configurations and how they are experienced and perceived by patients in the context of transitioning from hospital to home. It has been well established that many patients do not have an FP or other designated primary care provider [11–13]. In Canada, for example, in some jurisdictions, up to 15 % of the population do not have a regular FP [14]. Moreover, those without an FP are often the ones who need coordinated care the most [15, 16]. Also well-established is that FPs and specialists play variable roles [17, 18] and, at times, the roles traditionally played by the FP can be taken up by specialists [19]. Finally, it is also clear that collaboration and communication between providers frequently lack coordination [20, 21].

Mapping a patient’s network of providers can be challenging. Methods for doing so have used both quantitative and qualitative approaches [22–24]. Most approaches, however, are labour intensive and offer different types of insights. As a result, to date, such mapping exercises have been done in limited contexts. One study showed that greater centrality of providers in the network contributed to enhanced disease monitoring in patients with type 2 diabetes or heart failure [23]. Another study used in depth interviews to map out heart failure care teams, focussing on their inner workings from both a patient and provider point of view [22]. More common are studies exploring how patients with different primary care arrangements utilize health care resources in the community. For instance, patients without a regular FP and those who encounter access barriers in visiting an FP are more likely to use walk-in clinics [25–27], frequent the Emergency Department (ED), [28–30] and be admitted to hospital [31, 32]. Moreover, affiliation with a collaborative primary care structure or primary care team has been associated with lower rates of emergency visits and hospital admissions in some studies [33–36]. Less apparent, however, is what kinds of attachments patients with and without regular FPs have with other physicians such as specialists, other health care providers and how these relationships exist in relation to each other in their networks of care.

A deeper understanding of network configuration variability may support safer transitions of care back into the community. A patient admitted to an internal medicine service is likely to have multi-morbidity, [37, 38] present some degree of complexity in medical management [39, 40] and utilize many different health care resources outside of the hospital. Working through the nature of the attachments these patients have with different clinicians — in the context of a community network made up of social supports and other players — may help provide necessary information to make informed planning decisions during a hospital stay, and to not overlook certain realities about how patients navigate their health issues. By interviewing patients admitted to an internal medicine service at a teaching centre in Ontario, Canada, we set out to understand how they experienced and viewed these different provider relationships, particularly during transitions in care such as arising from declining health, hospital admission and discharge back into the community.

## Methods

### Study Design

Constructivist grounded theory was used to guide sampling, data collection and analysis [41, 42]. This methodology was felt to be the best choice because of its well established and rigorous processes for exploring complex social phenomena [41, 42]. Ethics approval was granted by Western University’s Health Sciences Research Ethics Board.

### Setting and Sample

From June 18th to August 22nd, 2019, 30 patients (aged 36 to 91, average & median age of 69.5, 17 male, 13 female) admitted to the inpatient internal medicine service of an academic hospital in London, Ontario, Canada were interviewed. During this data collection period, a member of the research team (L.P-S.) attended morning rounds several times per week with one of the three internal medicine teams at the hospital to identify potential patient-participants. Purposeful and theoretical sampling approaches were used to select participants with the intent of achieving maximum variability [41]. Initial sampling focused on identifying diversity in the number and types of physicians from whom patients received care. This was done by simply identifying admitted patients with multiple medical comorbidities (at least two chronic diseases) requiring longitudinal medical management^2^. As data collection progressed and insights were gained from the initial analysis, theoretical sampling was used to identify networks and relationships that were absent or underrepresented in the sample. For example, if multiple perspectives from patients with highly involved FPs had already been elicited, participation in physician team rounds and field observation could help identify potential patient-participants who relied more heavily on care from a specialist, or who were not rostered with an FP. In the context of the phenomenon being explored, there was an assumption that participants were consistently accessing care that was made available to them. Admitted patients with unique access barriers, such as those experiencing homelessness, or those with substance use or disorders or other social factors that contributed to a failure to access available care, were not sampled. Data were collected to the point of theoretical sufficiency – the point where the developing theory could be fully supported by the collected data and where sampling for discrepant cases failed to reveal novel insights [43]. Out of 23 patients approached who did not participate in the study, four actually refused due primarily to lack of interest, while others expressed interest in potentially participating at a later time. In the latter group, a mutually convenient time for participating in the study could not be scheduled prior to their discharge.

### Data Collection

Following rounds, with permission from the attending physician, patients were approached by L.P.-S. or J.T. and invited to participate in the study. Patients were typically approached and interviewed the same day (and no longer than one week after) they were identified. Researchers introduced themselves to the patient, explained their role, provided an overview of the study and its goals, and provided a letter of information and consent form which patients could opt to read themselves or have this reviewed in detail with the researcher. Family members, when present, were also invited to participate. Consented participants and their family members took part in an audio-recorded interview conducted by L.P.-S. at the bedside. Interviews were often conducted later in the day so as to minimize interruptions from hospital staff, and necessary discretion was used for participants sharing a room with another patient. Interviews were 20 to 60 min in length (mean length of 36 min) and semi-structured in nature. Questions and prompts^3^ focused on exploring participants’ different physicians and their perceptions and experiences with each over time and as they experienced health events such as a new disease diagnosis, current and previous hospitalizations and eventual discharge, or a significant decline in their health or functional ability. As we gained insight into patient perspectives of their networks, we modified our probes to more explicitly explore membership in the network of allied health practitioners, other community resources, family members and friends. During the interview, a visual sketch was created of the described network. Post-interview, with consent from the participants, a retrospective chart review^4^ was carried out to clarify details arising from the interview.

### Data Analysis

**Fig. 1:**
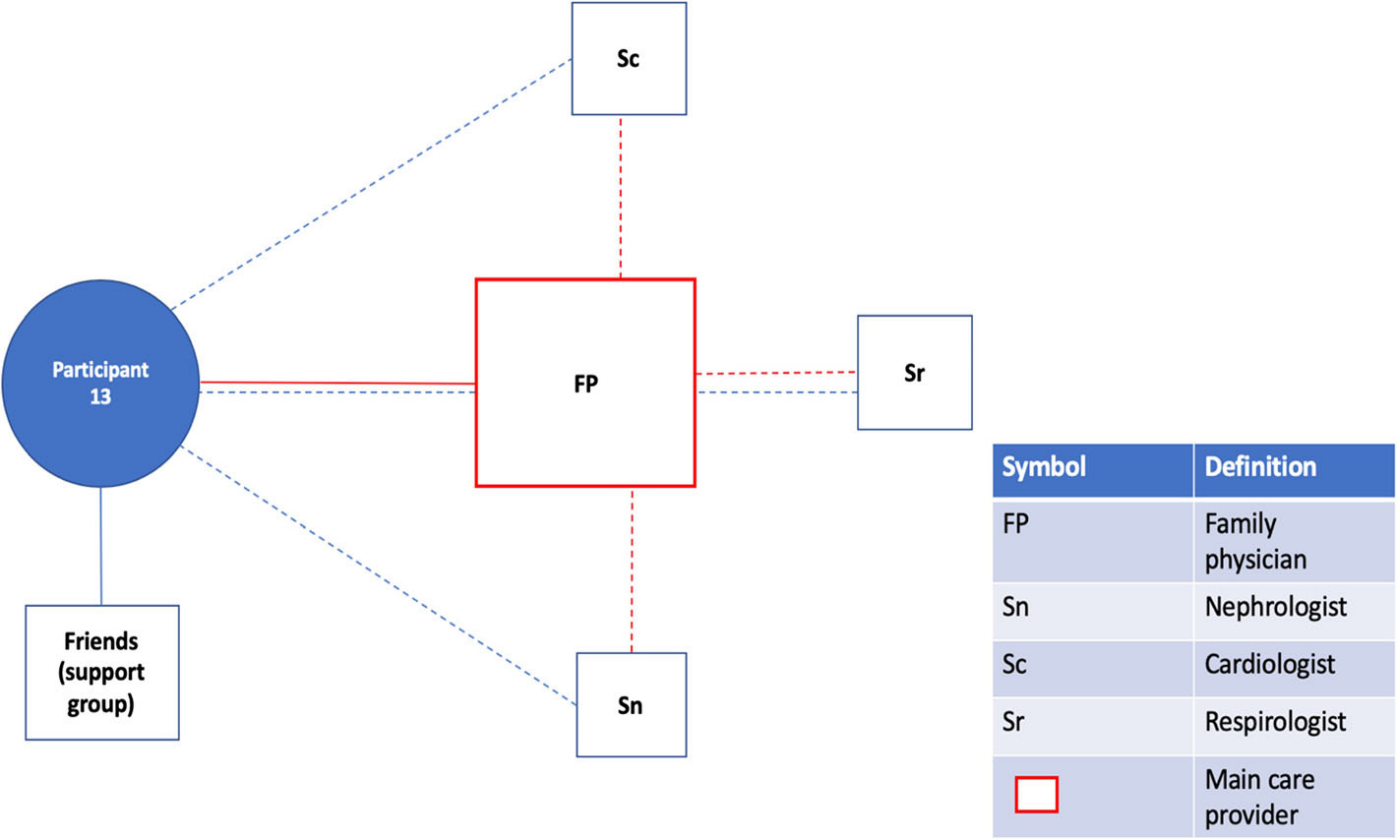
Patient Network of Care Diagram. A diagram such as this one was created for each study participant using information gained from the bedside interview. If a main care provider could be identified, they were displayed on the network diagram in red. For formally rostered patients, it was not assumed that the main care provider was their FP. Instead, the main care provider was the one the participants saw as the key care figure in their everyday life, or the one providing services of greatest importance to them. Distances from the patient to the provider were used as representations for the frequency of appointments with the provider. These were estimated based on direct questions about how frequently each provider was seen. A similar logic was used to dictate the size of the box for each provider, representing the relative importance of the provider to the patient and their network of care. Lines were used to connect patients to providers and providers to providers. Solid lines indicated relationships between patients and providers that had a personal dimension to them (patient and provider knew one another). In contrast, dashed lines between patients and providers indicated the provider was either an entity such as a hospital or where patient and provider were very unlikely to have a personal relationship based on the nature of the association (e.g. one-time specialist consultation). Dashed lines between providers indicated the existence of patient-focused communication between them. Patient-focused communication involving the main care provider was once again captured in red. Lastly, social supports were included in the network of care diagram

Analysis and collection took place in iterative cycles. Initially, interview transcripts were coded line-by-line (L.P.-S. and J.T.) using NVivo 12 (QSR, Doncaster, Australia)[44] and the sketch of the participant’s network of care made during the interview was reconciled from chart data. Regular meetings with members of the research team (M.G., J.T., L.P.-S.) were held to establish focused codes and review network diagram sketches. Partway into data collection, we recognized that producing high-level network diagrams accompanied by rich descriptions was a superior form of analysis for the data being collected (Fig. 1). These focussed accounts of participants’ narratives were synthesized from interview transcripts and help provide context for the network diagram. Examples of these rich descriptions can be found in Appendix B. Interviews evolved to reflect this enhanced focus on network mapping. Researchers M.M. and A.A. were brought into larger team meetings to assist with theoretical coding of network diagrams. Diagrams, rich descriptions, and transcripts were coded in multiple iterations using constant comparison. These codes were then grouped and categorized, stratifying participants along various levels of specific network of care attributes. Common archetypes of network configurations could then be identified following participant stratification within this established framework. This allowed for further exploration into the roles health care providers and social supports played within these diverse network types.

## Results

### Overview

We identified a set of network configurations to represent how patients perceive and interact with the health care system (Table 1). The description of these configurations starts with whether or not the patient is formally rostered^5^ with an FP. It then incorporates their relationship with their specialist(s), their social support system and their capacity for self-advocacy and self-care. Each physician relationship is further described along a spectrum from optimal to suboptimal. Differences in participants’ capacity or willingness to self-advocate, and the presence or ability of family or close friends to support them, allowed for further stratification.

**Table 1:** Final Network of Care Configurations for Study Participants

Formal attachment with family physician	Perceived relationship with family physician	Perceived relationship with specialist(s)	Social support system	Capacity for self-advocacy and self-care	Participant ID
**Rostered family physician**	Optimal family physician involvement	Optimal specialist involvement	Strong social support system	Independent	1, 5, 18
				Dependent	22
			Weak social support system	Independent	19, 21
				Dependent	10, 24
		Suboptimal specialist involvement	Strong social support system	Independent	
				Dependent	6, 17
			Weak social support system	Independent	13
				Dependent	27
		Specialist involvement played minimal role	Strong social support system	Independent	
				Dependent	16
			Weak social support system	Independent	11
				Dependent	
	Suboptimal family physician involvement	Optimal specialist involvement	Strong social support system	Independent	15, 28
				Dependent	4
			Weak social support system	Independent	31
				Dependent	7, 26
		Suboptimal specialist involvement	Strong social support system	Independent	25
				Dependent	8, 14
			Weak social support system	Independent	2, 23
				Dependent	
		Specialist involvement played minimal role	Strong social support system	Independent	
				Dependent	
			Weak social support system	Independent	
				Dependent	
**No rostered family physician**	Increased reliance on other services	Optimal specialist Involvement	Strong social support system	Independent	29
				Dependent	
			Weak social support system	Independent	20
				Dependent	
		Suboptimal specialist involvement	Strong social support system	Independent	3
				Dependent	
			Weak social support system	Independent	
				Dependent	
		Specialist involvement played minimal role	Strong social support system	Independent	
				Dependent	
			Weak social support system	Independent	12, 30
				Dependent	

### Physician Relationships

Participants could either be rostered or not rostered to an FP. If they were rostered, the nature of the FP’s involvement within the participant’s network of care – as perceived by the participant – could be classified as optimal or suboptimal. Participant perceptions of involvement appeared to be heavily influenced by how their FP helped them navigate recent changes in their health. This may have involved their availability for more frequent visits, opening new lines of communication, support with transitioning home from hospital, or their effectiveness in involving new secondary providers in the network (Table 2). We also identified network of care attributes common in participants not rostered to an FP (Table 2).

**Table 2:** Characteristics of optimal and suboptimal network components

Network of care components			
Family physician	Specialist	Personal supports	Personal capacities
**Optimal**	**Optimal**	**Strong**	**Independent**
Retains or further develops central figure/patient advocate and health care system navigator role Strategies for maintaining involvement in care of the medically complex patient: • Communicate with patient’s other physicians • Coordinate timely referrals • Offer personal line of contact with patient • Check in on them while in hospital • Set appointments soon after discharge • Advise them not to visit walk-in clinics and recommend avenues for after-hours care • Provide education materials • Enlist them in a paramedic home support program	Continuity over time Regular appointments Direct line of contact with patient Availability on relatively short notice Effective communication with patient’s other physicians May assume a Main Care Provider role for the period where they are being frequently seen Sees specialist(s) for health problem(s) that are of most relevance to them, or for health problems that are directly related to their recent hospitalization(s) or health decline	Has one or more people in the network who are actively involved/informed about all facets of their care One or more members can attend appointments/meet with clinical teams when hospitalized and as needed, advocate for patient Ideally have multiple layers; family members, friends, and/or neighbors When required, a coordinated effort involving multiple family members/friends takes place to offer support	Tends to comfortably advocate for themselves in hospital/health care settings Self-sufficient to a degree and able to adapt to changing circumstances, proactive Has a strong grasp of their issues/limitations and the type of support they require
**Suboptimal**	**Suboptimal**	**Weak**	**Dependent**
Does not play central health care figure role; may provide prescription renewal and offer episodic care for minor ailments Defers all decision making regarding major medical problems to other health care providers Does not appear to make effort to stay involved when specialists take on more central roles (e.g. heart failure or cancer care) Unable to offer timely appointments when health deteriorates or following transition home from hospital	Perceived investment in their health and well-being is minimal Lack of continuity over time (e.g. group practice where the same specialist is rarely seen) Intervals between appointments feel too long/rationale for intervals is not clear Unclear communication with patient regarding role in care Communication with patient’s other physicians perceived as poor Specialist no longer easily accessible or connected to network (e.g. works in a different city where the patient used to live)	Not present – participant does not have a strong individual or network of family/friends who can offer support when needed Not able – family/friends they do have cannot/will not invest the time and effort required to advocate for or support them meaningfully	Poor self-advocacy skills; may be related to social determinants of health including level of education Disengaged or disinterested in trying to improve health and well-being Denial about severity of health issues Impaired ability to self-advocate: mental health, substance use disorder, cognitive impairment
**No Rostered Family Physician**	**Playing a Minimal Role**		
Increased reliance on other services: • Walk-in clinics • Emergency Department/Emergency Medical Services/Urgent Care • Homecare • Caregivers	Specialist not a relevant/contributing component of the patient’s network of care providers presently and in last two to three years Few specialist referrals in recent years, which were of little perceived value to the patient		

Regardless of the presence and nature of involvement of an FP, participants could also be classified based on the roles they perceived specialists played in their health care. This involvement similarly existed along a spectrum of suboptimal to optimal but could also be perceived as having played a minimal role. Not uncommonly, there was more than one specialist involved in the patient’s network of care; to keep Table 1 manageable, these are not fully depicted. The nature of these relationships could resemble that of a main care provider, be entirely consultative or fall somewhere in between. What each participant perceived they needed from specialists differed; we used their stories and experiences to determine whether their involvement had been optimal or suboptimal. Regardless of type of physician (FP or specialist), from the participants’ perspective, “suboptimal” referred less to any one characteristic and more to the extent to which a particular characteristic’s absence was perceived as essential.

### Different Physician Configurations

Physician involvement within patients’ networks of care providers typically formed the scaffold for the final configurations of a network. Certain models of physician involvement appeared frequently within our sample, and participants who shared them echoed one another’s experiences and sentiments with navigating illness and their network of care providers. Whereas some configurations were described as optimal across the network, others were perceived as highly ineffective. For some patients, even having one optimal relationship could compensate for other, less effective ones. Furthermore, to be considered optimal or suboptimal, participants did not require that physicians display all characteristics listed in Table 2. For example, the FP of participant 26 was considered suboptimal, primarily based on their inability to offer timely appointments.

In the sections below, we describe each of these representative network types in more detail. Supporting quotes from participants who endorsed these networks are found in Table 3 and are indicated in the text within parentheses as ‘Q’ followed by one or a range of numbers which denotes the quote number in Table 3, and ‘P’ followed by one or a range of numbers which denotes the participant IDs for said quotes.

**Table 3:** Representative and illustrative quotations (as referred to in text)

Quote #	Quote	Patient ID	Illustrative Concept
1.	I: So, between the different doctors that you see, you get a sense that your family doctor is the main care provider?R: I think I would say that, yeah. He’s pretty good at keeping in touch with people. When I see him, he’s quite likely to say, “Oh, yes, I talked to [general surgeon] about you,” and so on.	1	Main care provider communicating with participant’s other physicians.
2.	R: So [family physician] wanted all the specialists…. And this is the only way she could get the specialists together, to put me in this [paramedic] program. Because through that, she could get all that information. Otherwise, forget it.	13	Main care provider communicating with participant’s other physicians.
3.	R: Well, I didn’t bring it up with anybody. I just complained about it, you know. But [family doctor] sent me to a specialist one time. I thought I had a bowel problem. And she said… She’s sharp, you know. She said, “No, I think you’ve got something else.” So she sent me to Dr. [name], who’s a heart specialist. And I found out I had this PAH [pulmonary arterial hypertension].	21	Main care provider providing timely and necessary referrals with specialists.
4.	R: Maybe 2 months.I: To see your family doctor?R: Yeah.I: Is that a concern for you?R: Yeah, when you really need them. But the receptionist just tells you to go to a walk-in clinic.	26	Lack of engagement from main care provider.
5.	R: Well, what he would do is he would give me a form for blood work. I’d go get the blood work done. And I’d come in and I’d say, “[Family physician], the feet are still swollen.” And he’d go like this and he would say, “Yup, that’s water all right. What are we going to do about that?” And you know, there would be a tweak or a change or a new pill or something to try to control that.I: But it never did much?R: It didn’t do much in that it began to change. Well, it got worse.	2	Main care provider not proactive bringing other providers into participant’s network of care.
6.	I: So you said that your rheumatologist is actually the one that you saw as your main care provider prior to coming here.R: Pretty much, you know.I: So could you phone them if you had a concern?R: Yes. They actually have phoned the house to see how I am.	5	Specialist stepping into main care provider role.
7.	I: …since you’ve been deferring to specialists for those issues, when you do see your family doctor, it’s just a routine health visit?R: Yeah, usually something that’s more mundane. You know, I’ve got a rash, you know, I’m this or that.	28	Family physician’s role changes after specialist steps into main care provider role.
8.	R: I get blood drawn. [Nephrologist] does like you guys do, he checks the heart, checks the lungs. And he tries to go through my back history, especially for other doctors. Like for [hypertension specialist], what kind of medications he’s put me on, and if they need adjustment or not.I: And that’s every month?R: Every month.	4	Strongly engaged specialist acting in a main care provider role.
9.	I: So back to the family doctor, what would you say is her level of involvement or investment in your overall care?R: Really not that much actually. She’s only in her office two days a week. And usually when she sees me, like I said, if she doesn’t know how to diagnose me then she sends me somewhere else.	4	Family physician’s role in care is limited in the context of specialists providing the majority of care.
10.	I: Is there one that you consider more to be your main care provider at this point, the one that’s most invested in your health that you’re getting the most care from?R: I would say the diabetic care team.I: Okay. And so your family doctor is more so coordinating the other aspects of your care?R: Yeah. And that’s on his own terms because he said that there’s no point in getting too many cooks in the kitchen. So he’s happy with what they’re doing over there. So as long as he can see what they’re doing, knock yourself out.	31	Specialist(s) are strongly engaged and providing majority of care, FP’s role in care is limited.
11.	R: There’s three of us. There’s actually four kids. One is in Alberta and the other 3 are here. So we’re all involved in her… we all do something different for my mom. But it all comes together. So my one brother lives with her. But he’s feeling a little bit overwhelmed. My one brother lives in Aylmer. And he also does things for her. I take her to appointments, get her fresh groceries. He gets her the Costco run. So you know, banking and stuff. We kind of all do our thing as far as when she needs to go somewhere. If I can’t do it, my brother will. We’ll tag team. He’ll drop her off, and I’ll be there. And then I’ll take her home. So it’s kind of working. But we’re at a stage where I think something else needs to happen.	17 (daughter)	Social supports mobilizing around the participant to provide the necessary care in the face of participant’s declining health and function.
12.	R: So I’ve been on Amazon and the grocery delivery. And I figure between that and that… I get my Amazon things. An Amazon rollator delivered to my door. And I bought a bath bench for my shower. Just a little shorty. Not that big thing. And I got that on Sunday, and I set it up on Monday. And I came in here Monday night. That’s in case I have…I don’t feel strong enough to stand. I can just sit there. It’s just a nice little simple bench.	23	Participant with limited social supports finding ways to adapt and retain independence in the face of worsening health and functional decline.
13.	R: Take my blood pressure? Nothing like that, no. He goes in and he says, “Do you need anything?”. I’ll ask him for the results and stuff like that. And it’s all in and out within 10, 15 min. He doesn’t take much time to sit down to talk to me.I: And does he… Because he must know that you have Brugada, that you have chronic pancreatitis.R: um-hum.I: Does he say… Like he’s deferring to the specialists for opinions on those problems?R: Yeah, he tells me to call them.	7	Frustrations of the socially isolated participant at the level of primary care.
14.	I: I understand. So that’s [non-emergency patient transport service] a service that picks you up where you live and then takes you back afterwards?R: Yes. Well, you have to make appointments 3 days ahead of it. You have to have the hour and you have to have also kind of know what time you’re going back. And it doesn’t always work out. If I go to my doctor, and say it’s 12:00. And I think maybe 2:00 but it might be 3:00 before I’m finished. You see, that doesn’t always work out.I: Oh, I see. So you have to do it a couple of days in advance, and then plan a time that’s going to work for them and for you.R: Yeah. And of course a taxi. It’s getting too expensive.	10	Frustrations of the socially isolated participant with regards to transportation to medical appointments.
15.	I: And on the personal end, do you have somebody that helps take care of you or helps drive you to appointments or anything like that?R: No. I do pretty much most of it myself. When I don’t feel like driving or just don’t think it would be a good idea for me to be driving, I’ll take a [taxi].I: Got it. So you manage pretty well on your own?R: Yeah. Well, I’ve still got a pretty good brain.I: Fair enough. Do you have any home care services or anything like that? Somebody coming in to help with anything?R: Yeah. I have PSW workers through St. Elizabeth that come in every morning for an hour and a half.I: Okay. What kind of things are they helping with?R: Well, personal hygiene, getting dressed, stuff like that.	27	Formal in-home care services becoming an essential aspect of the socially isolated participant’s network of care.
16.	I: And if you have a health concern that comes up, are you phoning the doctor’s office, are you going to emerg? What do you typically do?R: It depends on how serious it is.I: Okay. So, let’s talk about the last 2 or 3 years, what are the things that have come up, if any?R: I can’t remember, there’s just been so many things. But when things get severe enough, I just call an ambulance and get taken to the hospital.	27	Overt reliance on emergency medical services and the hospital in the socially isolated patient during a disease exacerbation.

#### Optimal family physician with different specialists involved

Some participants described benefitting from a highly engaged FP who coordinated timely referrals and was in close communication with the participant’s other physicians (Fig. 2a). The involvement of the FP created a more collaborative and organized network of health care professionals, which participants described as optimal (Q1-3;P1,13,21). Having this degree of provider involvement at the primary care level often took the emphasis for continuity of care off of specialists, who could then sufficiently complement the participant’s network of care acting in supporting and consultative roles.

**Fig. 2a:**
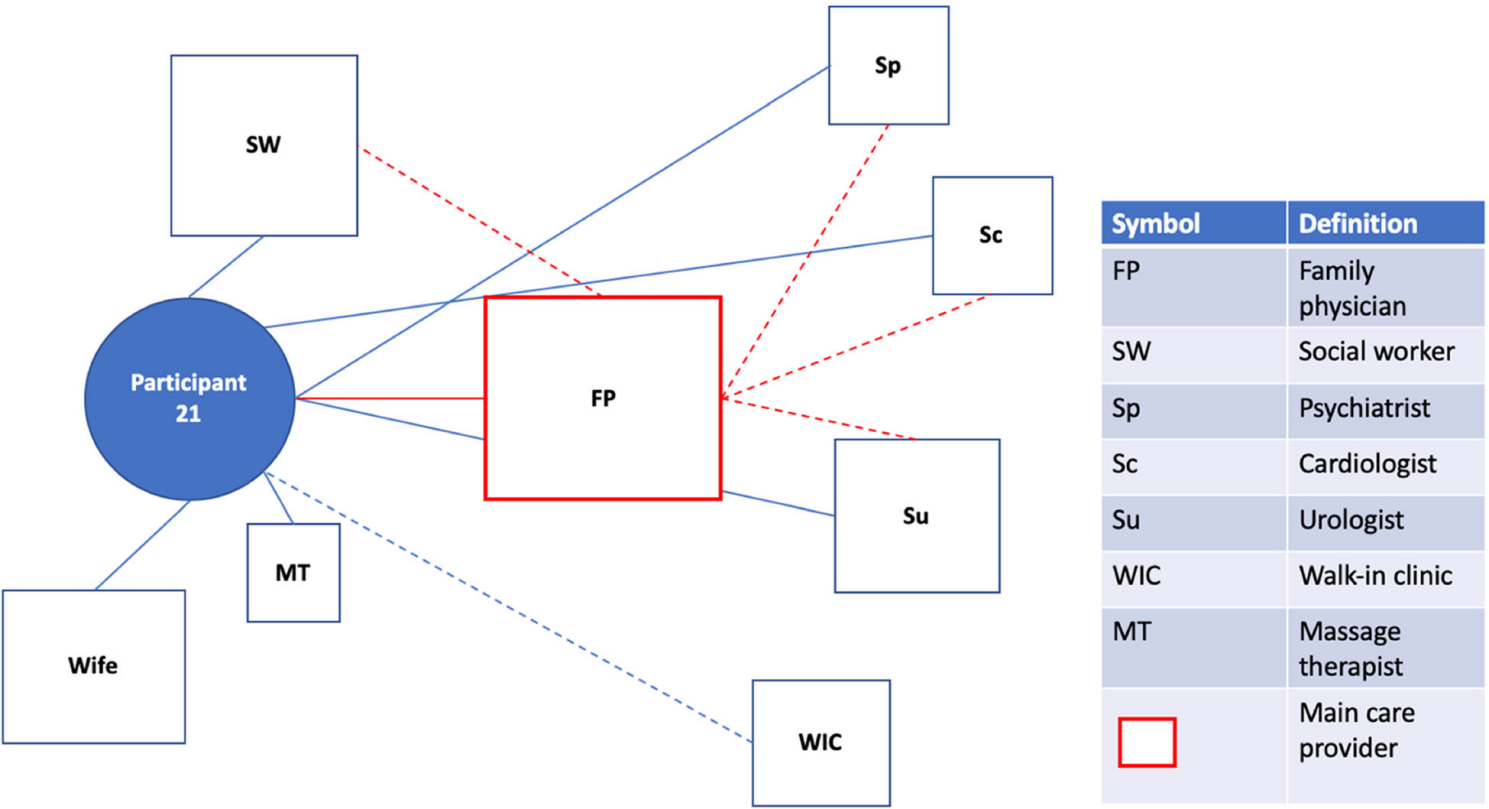
Participant 21. The FP is heavily involved in patient care, acting as the main care provider within this network. Involvement of specialists and a social worker arose from referrals made by the FP, who remains informed about care received from these other providers longitudinally. The cardiologist involvement, in particular, is recent and reflects the FP’s effectiveness in bringing another provider into the network for a new health problem (pulmonary hypertension)

#### Suboptimal family physician with different specialists involved

Some participants had care provider networks that were visually similar to those with an optimally engaged FP and various specialists. These participants, however, did not experience primary care the same way, attributable in part to a perceived suboptimal FP. While the FP was still the most central health care figure in their network of care (Fig. 2b), participants did not endorse this provider adequately supporting them. This included difficulties making timely appointments (Q4;P26), obtaining referrals (Q5;P2), and concerns regarding the quality of care. Participants with a perceived suboptimal FP described looking for alternative care providers to support them, such as visits to the ED, walk-in clinics and referrals to specialists.

**Fig. 2b:**
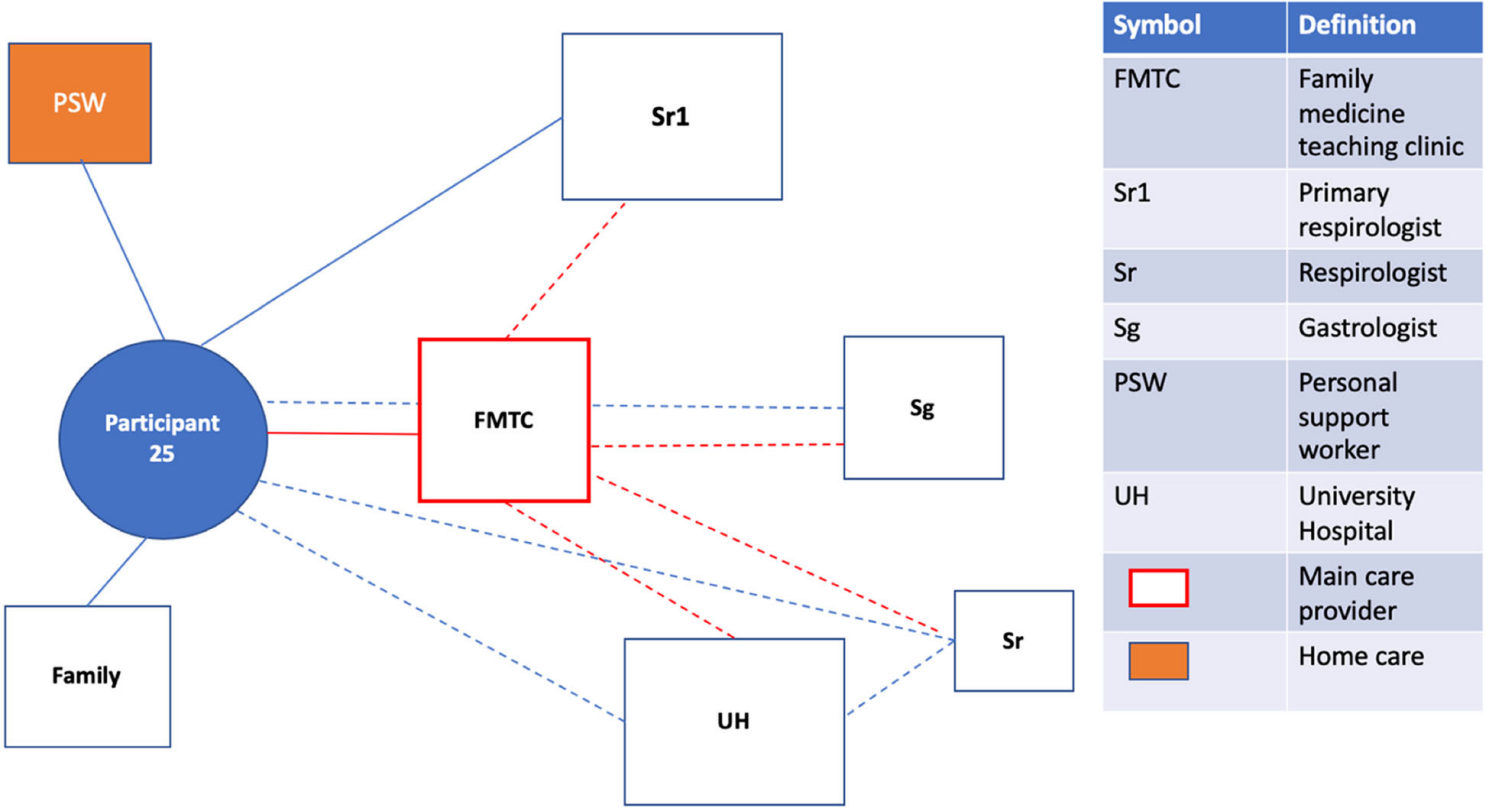
Participant 25. The FP operates out of a teaching clinic, where the patient may see a resident, clerk or nurse practitioner at any given appointment (unless they request to see the FP, in which case they must wait weeks). While there are no issues regarding formal communication and relay of visit notes between the clinic and the patient’s specialists, the patient is not clear about who is to provide their main COPD care: their respirologist or FP. The patient feels that not being able to see the FP themselves consistently contributes to this lack of clarity. When at their clinic, the most consistent message is to defer to their respirologist for COPD management. By contrast, the respirologist only wants to see them following acute events. This patient has had numerous hospitalizations due to COPD exacerbations, and the hospital has become an important provider in their network of care

#### Strongly engaged specialist (temporary), variable family physician

Some participants described the increased involvement of a specialist following a new disease diagnosis. In many cases, the specialist transiently became the main care provider, especially in the absence of other complex health issues (Fig. 2c). The specialist acted in this central role optimally for some participants, and sub-optimally for others. When present, the FP typically saw at least some aspects of care become the specialist’s responsibility and maintained variable levels of involvement in patient care (Q6-7;P5,28).

**Fig. 2c:**
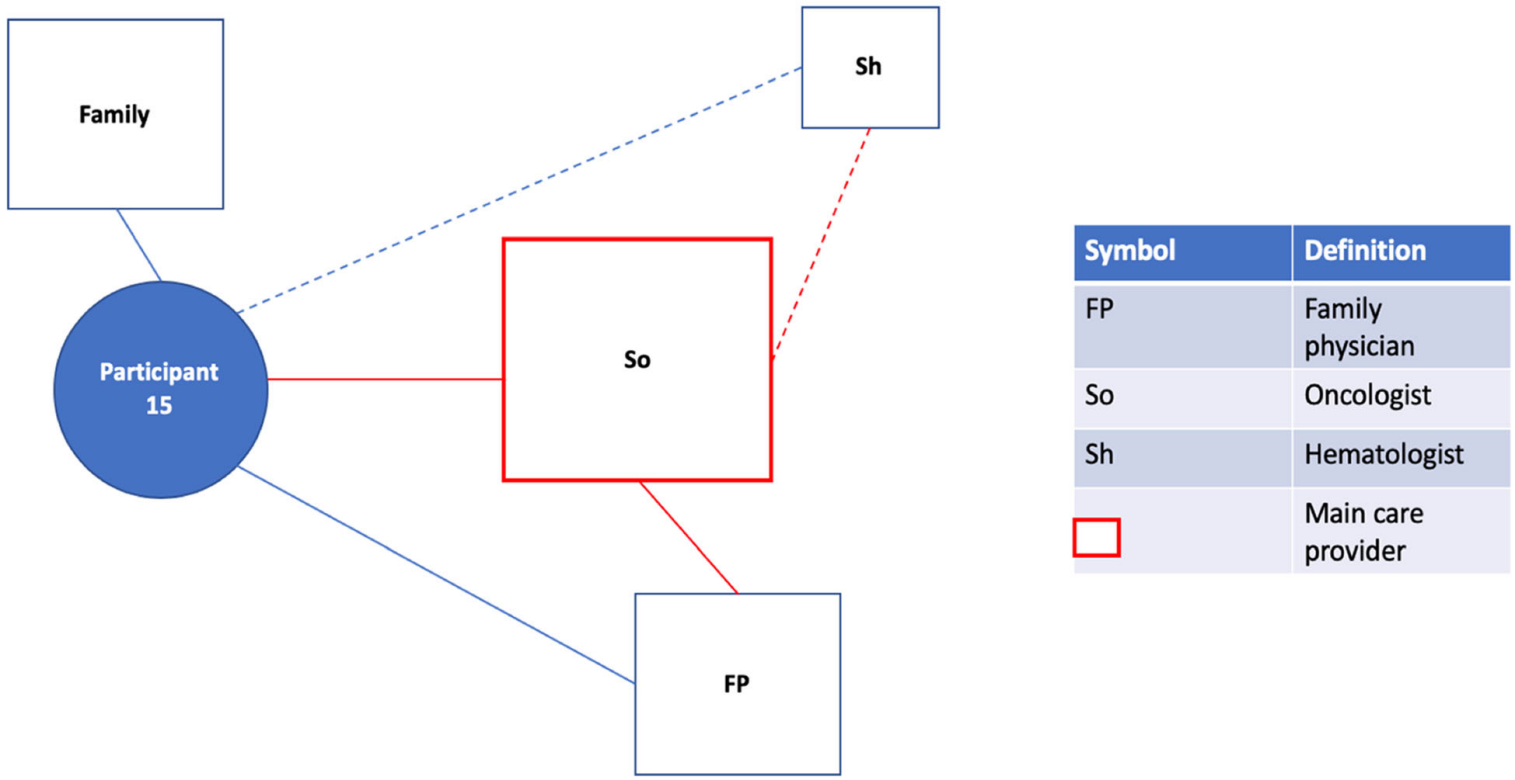
Participant 15. Following a diagnosis of follicular lymphoma, the previously healthy patient, who only occasionally visited an FP, saw an oncologist become the main care provider and central figure in their network. The oncologist is in regular communication with other providers such as the FP and with a hematologist who was brought into the network and performed an eventual transplant procedure. The oncologist’s temporary role as main care provider was also highlighted by an incident where the patient went to the ED suspecting they had a blood clot. It was this individual that followed up and was in communication with the hospital to determine what had happened

#### Strongly engaged specialist(s), less engaged family physician

Some participants, by virtue of a chronic health condition, had experienced a specialist acting as their main care provider for a longer period (Fig. 2d). When present, the FP’s involvement was limited to caring for minor ailments and making referrals unrelated to the condition for which care from a specialist had been sought. Participants often viewed the frequent and regular care received from this specialist as optimal. However, difficulties relating to coordination between providers were frequently experienced when a specialist and not an FP acted as the central figure, which led to overall frustrations with the network of care. Other participants found themselves relying heavily on several specialists who they saw for management of various chronic conditions. For these participants, FPs were similarly seen less frequently and had little to no role in the participant’s chronic active issues (Q8-10;P4,31).

**Fig. 2d:**
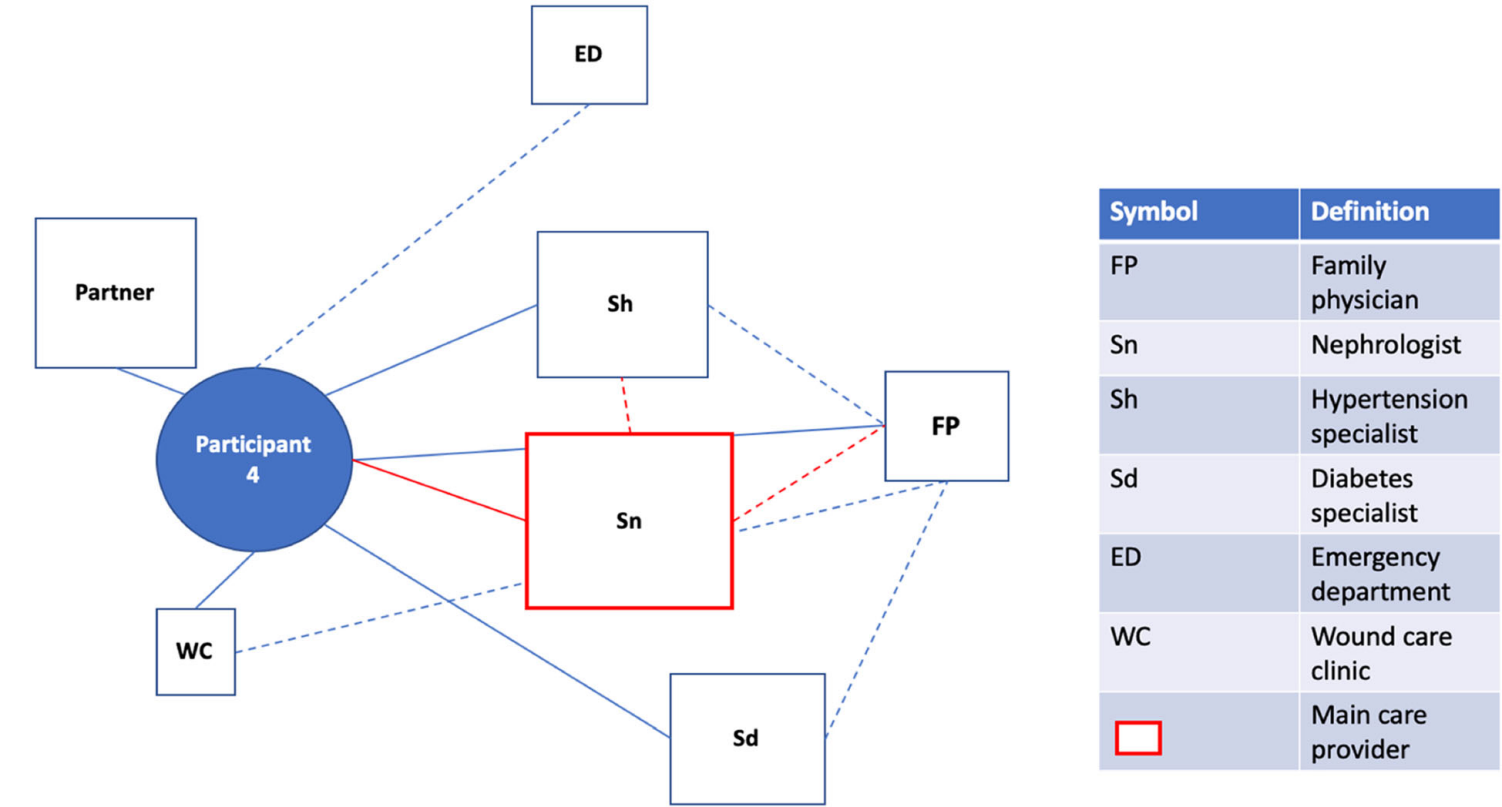
Participant 4. This patient sees their nephrologist as their main care provider, despite having an FP and being attached to other specialists who are also seen regularly. The patient feels that this specialist is the most involved in their care. Furthermore, the nephrologist’s work revolves around being informed about care received from the patient’s other physicians (e.g., knowing what medications have been prescribed by the hypertension specialist and ensuring these will not worsen kidney function), reinforcing their role as the most central provider. The FP’s role is limited in this particular network to a very peripheral role, being seen far less frequently than the patient’s other physicians. Of note, the patient feels their network lacks centrality and a true team-based approach

### Social Supports and Personal Capacities

Important differences also arose between patient networks with similar physician scaffolds based on participants’ social supports and personal capacities. As shown in Fig. 2, participants described different levels of involvement from family members and friends in their network of care. When present, strong social supports enabled participants to adapt to challenging circumstances. One participant was a widowed 89-year-old woman with very limited mobility, several chronic health problems, including congestive heart failure, and limited English. She still lived in her home at the time of the interview, likely due to her three children’s tremendous, coordinated support. All three children were frequent visitors to the hospital, engaging with staff, advocating for their mother and ensuring she was still included in conversations about her care (Q11;P17). In participants where a degree of social isolation was observed, or where care provided through social supports was inadequate, there appeared to be a greater requirement for personal capacities. As seen with one participant, even when social supports were absent and physician involvement was suboptimal, the capacity for self-advocacy could play an important role in navigating periods of illness or functional decline (Q17; P23).

Participants who lacked strong social supports and who appeared to be less capable of self-advocacy expressed difficulties navigating their network of care. This could include frustrations at the level of primary care (Q13;P7) and issues with transportation to medical appointments (Q14;P10). Furthermore, hospital admissions, ED visits and more intensive assisted living support appeared to play important roles in network of care diagrams for participants who lacked strong social supports and who appeared to be less capable of self-advocacy. One participant (P16), a 71-year-old male, had had numerous hospital admissions related to excessive alcohol use over the last two months before being interviewed. He had lost his wife months earlier, and aside from an FP (whose involvement in care was perceived as minimal), he had just one friend in the way of community supports. In this context, the hospital quickly became his main care provider. Other participants lacking adequate social supports described heavy reliance on personal support workers and other formal home care arrangements to live independently (Q15;P27). They also described an increased reliance on ambulance services and perceived the ED and hospital as key health care providers in their network. (Q16;P27).

## Discussion

We have identified multiple networks of care configurations in a typical internal medicine patient population worthy of further reflection. By interviewing patients on the wards, we were able to make visible the importance of care provider networks in subsequent care and how to determine which patients require special consideration. We have shown that the most salient factors in differentiating how complex patients experience navigating their network of care are: the presence of and nature of involvement of the FP and any specialist(s) in the network. While we did not initially set out to explore this, we also identified that the collective strength of their personal support system and their degree of independence and capacity also appeared to play a pivotal role. Below, we discuss implications in relation to a few key areas: discharge transition; central provider(s) (FP and/or other specialty) and the variability in patient and social support.

This study provides important insight into the various network configurations that might be found and the types of questions that should be explored with each admitted patient. Specifically, we would argue that understanding which type of network a patient exists in is essential when planning their effective transition back into the community. Discharge planning is an important component of transitional care and can have considerable impact on morbidity, mortality and rates of 30 day rehospitalization [45–47]. In particular, deficits in coordination of care between hospitalists and community providers are pervasive, and are associated with adverse clinical outcomes and unnecessary health care utilization [47–50]. Similar to findings from Kiran et al. (2020), participants who had relied more heavily on formal services established during prior hospitalizations (e.g. community services organized by the hospital team) described frustration due to the transient and unpredictable nature of these arrangements [51]. By contrast, those describing an optimal central relationship felt that they were best suited to organizing more durable community supports, meaningfully involve other clinicians and make clinical decisions that integrate information from multiple sources.

Incorporating approaches to identify and better understand a patient’s networks of care may therefore offer a novel approach for improving safety at the time of discharge and can build upon other research flagging the importance of care coordination [20, 21]. We would argue that, while much has been written about the importance of communicating with a patient’s FP prior to discharge and through the discharge summary, [7, 48, 52, 53] there is a need to broaden this to include specialists, non-physician health care providers and social supports who play an important or central role. In some cases, this may be one individual and in others, it may be a combination. To date, this issue has been underexplored and inadequately advocated for in relation to improving discharge transition safety.

Having a provider who played a central role in the network, regardless of specialty, appeared to be essential. Consistent with prior research, [21, 54, 55] patients felt best served when they had a highly involved FP at the centre of their network. Those who did not have a physician who they viewed as central and optimal, described a very different experience of care over time. This appeared to be equally true for those with minimal or no providers, and those who were technically rostered to an FP and/or had numerous specialists involved in their care if they perceived there was no collective coherence to their care. Prior studies have shown that not being rostered to an FP can negatively influence readmission and health care resource utilization, [30, 56] as can not having a regular FP [32]. But what about those with a different central care provider? Although some participants did not have an FP – which is consistent with prior epidemiologic research [11–14] – other patients with an FP felt they did not play the role of central provider. Rather, they viewed one or more of their specialists as occupying this role. This phenomenon has been observed in other contexts [57–59]. In many jurisdictions [60–62] and in proposed stepwise models of care, [63–65] health care design is predicated around an FP or nurse practitioner playing the central role. While our findings and that of prior research support the excellence of these models, [21, 55] many of the alternative networks may be necessary – especially in locations where patients are unable to access a regular FP or a regular FP who plays an optimal role in their care [66] – and therefore need to be further explored. For example, the perception of having an optimal central provider who is a specialist does not necessarily mean that patients are receiving comprehensive primary care such as age-appropriate screening. Rather, its strength is related to the extent to which medical problems, perceived by patients as dominating their care needs, were being well addressed. For some, these relationships arose as a temporary centrality in relation to an acute condition requiring that specialist’s support over a period of time (e.g., cancer care or heart failure care), whereas for others, it was founded on a longer-term relationship and may have arisen in relation to an existing gap. Other researchers have also identified this phenomenon, [67–70] and it requires further exploration into how, where, and in which patients or patients with which kinds of primary and secondary care structures this arises. Failing to do so may lead to patients with non-conventional networks (especially those without an FP as the central provider) being excluded from healthcare innovation planning and opportunities arising from these.

Some of the patients we interviewed did not appear to have equal access to an optimal central provider and, consequently, had a greater need for a comprehensive transition plan. According to studies like those of Aoki et al. (2018) and Smith et al. (2009), this is not a surprising finding [71, 72]. While the Canadian health care system offers universal health insurance coverage, it in no way guarantees that all citizens receive equal care [25, 73]. In particular, patients with lower socioeconomic status, and those who struggle with mental health, dementia, social isolation, or substance use disorders are well recognized to be at risk of poorer quality care and outcomes [74–79]. We would also argue that their personal capacities and social supports’ ability to advocate played a further role. In the context of deteriorating health, patients who had neither described being more reliant on the hospital and having frequent ED visits and hospitalizations. Moreover, for some, there was the perception of a hospital visit as a routine occurrence, such that it became a significant component of their network of care. This has also been seen elsewhere [80, 81]. Numerous studies have tried to identify key patient factors that lead to higher risk of frequent ED visits and readmission [8, 82–84]. While they have identified risk features, they have not necessarily been able to consistently pinpoint which patients fall within these higher and lower risk categories [85]. In part, this may have to do with inadequate consideration of a patient’s network including their own capacities and personal supports. Going forward, we would argue that these need to be more consistently explored and that strategies need to be developed for supporting patients with inadequate networks. Promising considerations include enhanced post-discharge clinics [86–89] and patient navigators – individuals who assist with transitioning complex patients without a main care provider or formal advocate [90, 91]. Given the cost of such programs, strategies for consistently identifying patients in need of such services likely involve exploration of their networks.

Our study has several limitations that are important to note. First, the focus of a study of this type is transferability and not generalizability. Insights gained therefore need to be tempered by consideration of local contextual features. Consistent with our methodology, we also acknowledge the data as a co-construction between the research team and the data itself. One of the strengths of the study team however was the diversity of our team members and the theoretical triangulation that this brought to our considerations in sampling and interpretation. Other study-specific limitations include stopping data collection once theoretical sufficiency was achieved. As a result, we do not have specific examples from every type of identified network configuration. For example, we do not have any examples of patients where the central health care provider was a nurse practitioner. Finally, this study was not designed to determine the prevalence of different network configurations. Therefore, future research should explore both prevalence of networks and network differences in outcomes such as morbidity, mortality, utilization and cost.

## Conclusions

In conclusion, our findings have important implications for transitional care planning and health care design. We propose that all patients being admitted to an internal medicine inpatient team need to have their existing networks explored and taken into consideration in discharge planning. Doing so may face inertia, as this represents a paradigm shift in acute care. However, time spent exploring pre-existing patient care networks may be an opportunity for greater efficiency in the discharge process, as care needs would be more effectively triaged in favour of those with precarious arrangements, rather than solely on clinical need. Furthermore, we would suggest that a one-size fits all model of health care is unlikely to meet the needs of all patients and therefore, more work needs to be done exploring how to support patients where the FP does not play the role of central care provider and for those whose personal capacities and social networks lack an effective advocate. As part of this work, how practitioners communicate and negotiate their relationships with each other, and their patients, should likely also be explored.

## Supplementary Information

**Additional file 1:**

**Additional file 2:**

## Data Availability

The datasets analysed during the current study are not publicly available in order to protect participants’ anonymity but can be made available from the corresponding author upon reasonable request.

## Abbreviations

FP: Family physician
ED: Emergency department
COPD: Chronic obstructive pulmonary disease

## Footnotes

1 The literature on this topic uses many synonyms or near synonyms such as general practitioner or primary care physician. In Canada, primary care physicians are called general practitioners or family physicians. For the purpose of this paper, we will therefore stick with the term family physician when referencing this group of practitioners.

2 Examples of such comorbidities include heart failure, Diabetes Mellitus, and Chronic Obstructive Pulmonary Disease. Simple hypertriglyceridemia well controlled with medication, for instance, would not be considered as contributing to a patient’s comorbidities.

3 Questions and prompts were initially developed by the research team and followed a semi-structured format which would allow the interviewer to more freely explore participant perspectives, especially early in data collection. These prompts evolved and were fine-tuned as more interviews were conducted. The interview guide developed for this study is provided as Appendix A.

4 Since returning transcripts to patients for review was deemed difficult in the circumstances, a retrospective chart review was used to ensure that key details from the interview could be consolidated. This was especially helpful when participants were unsure of the name of a medical condition, of the type of specialist they had seen, or in other instances where patients recounted and experience but were unsure of details regarding its medical nature.

5 Rostering refers to a capitation-based approach to primary care in Canada where individuals are formally registered with one provider (typically an FP) by way of either choice or geography.
