# Supplementary material for: Discharging the complex patient - changing our focus to patients’ networks of care providers
Source: BMC Health Serv Res. 2021 Sep 10;21:950. doi: 10.1186/s12913-021-06841-2 (PMC8431846; doi:10.1186/s12913-021-06841-2)
Supplement: Supplementary file 1 — Additional file 1: [file 12913_2021_6841_MOESM1_ESM.docx]

**Appendix A:** Semi-Structured Participant Interview Guide

Synopsis: Our conversation is going to centre around what your network of care providers or circle of care looks like currently and in the past 2-3 years. I’d like to get a better understanding of who you see and why you see them, as well as any personal supports that you have.

- Who are the different health care providers that you see when you’re not in the hospital?
- Why do you see them?
- How often do you see them?
- How does it go when you see them? Can you describe a typical visit?
- How is the follow-up for issues that you bring up?
- How do you feel about the care you get from them?
- What would you change?
- How important is/are this individual/these visits for you?
- Who do you see as your main care provider? /Who is the most invested in your overall care?
- *mention that it doesn't need to be a person per say (could be a walk-in clinic if no rostered FP, Emergency Dept, a family health team, etc.)
- Why would you say it’s them?
- Who else are you going to see? Any specialists? Any allied health professionals such as dietitians, social workers, physiotherapists, people coming to your home?
- *Similar line of questioning – why do you see them, how often, etc.*
- Do they ever mention looking at the notes that the other doctor wrote in your chart, or does he/she ask you what another doctor had told you?
- Do they ever communicate about you as far as you know?
- Have you had any other hospitalizations in the past 2 or 3 years?
- What were they about?
- Did your family physician/other MCP communicate with you at all during or afterwards? How did they get involved?
- How do you feel about the care you received in hospital?
- Did you feel like any of what was done in hospital continue once you were discharged? Did the care you received from your doctor change afterwards?
- Were you connected to any other health services after that hospitalization?
- Who are the people that you can depend on?
- Family? Friends? Neighbors?
- What do they do?
- *if they tell you there is no one, ask about what they would do if something happened (e.g. in the home if they couldn’t walk and needed help, or something that relates to their particular health condition(s))
- Who can you call if you have a health concern or any kind?
- Does anyone come visit you in hospital?
- Do you ever go to walk in clinics? Urgent care? Emerg? – if so, why?
- Does your family doctor encourage you to go to walk-in clinics if they’re not available? Do they know that’s something you sometimes do?
- Why do you not see this individual or service anymore?
- Overall, before we end off, how do you feel about your network of care providers and their investment in your health in general?
- Is there anything you wanted to add?

Thank you for your time.
