# Supplementary material for: Discharging the complex patient - changing our focus to patients’ networks of care providers
Source: BMC Health Serv Res. 2021 Sep 10;21:950. doi: 10.1186/s12913-021-06841-2 (PMC8431846; doi:10.1186/s12913-021-06841-2)
Supplement: Supplementary file 2 — Additional file 2: [file 12913_2021_6841_MOESM2_ESM.docx]

**Appendix B:** Participant Network Diagrams and Interview Summary Examples


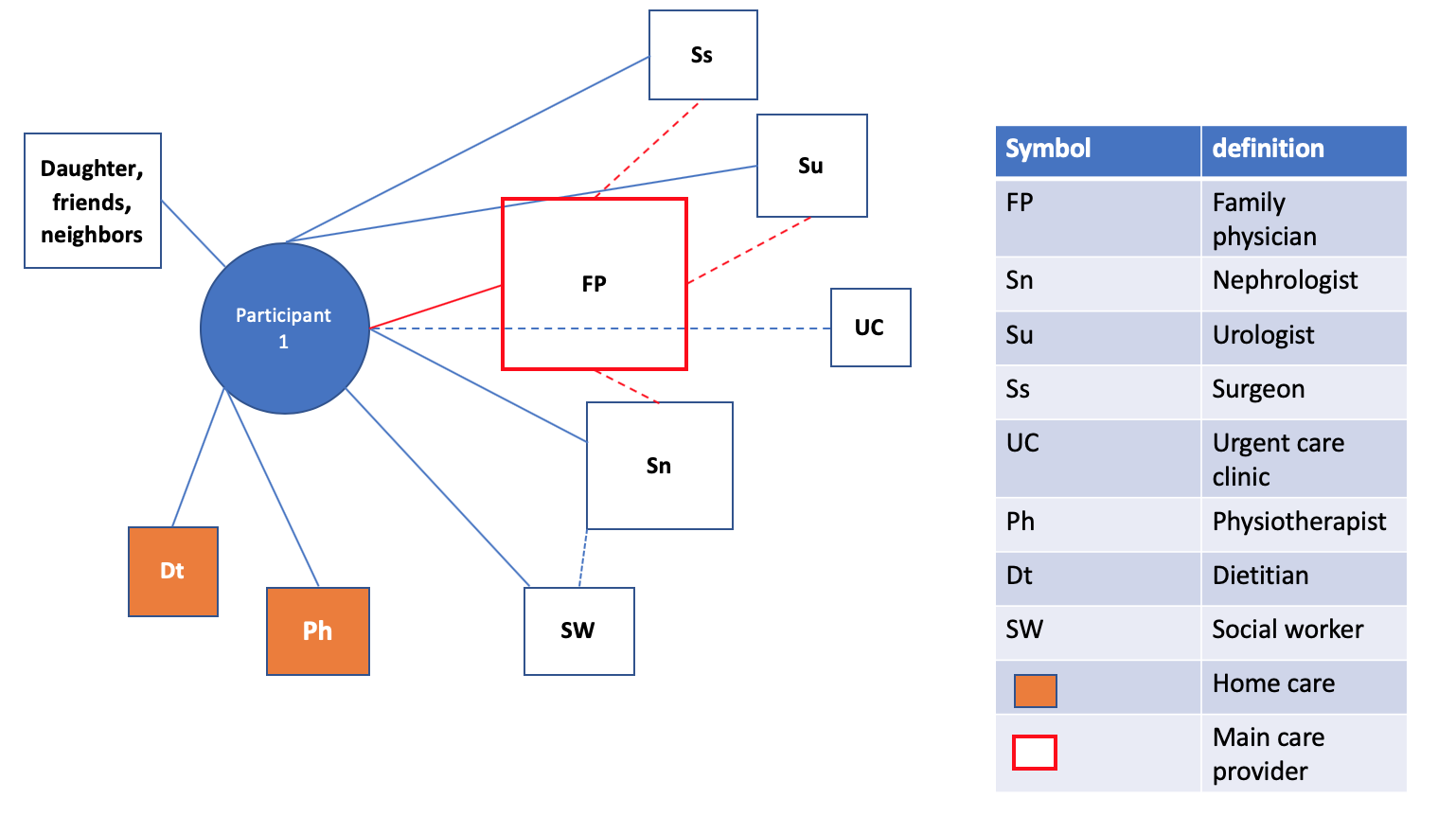


**Participant 1:** 90-year-old female who was admitted due to episodes of vertigo. Her active health issues include a high output colostomy bag which has led to episodes of dehydration, worsening kidney function and kidney stones (requiring placement of a nephrostomy tube), recurring bladder infections and a breast mass for which she had undergone a lumpectomy procedure. She considers her family physician (FP) to be her main care provider and has seen them every few weeks in recent months for the management of issues such as those arising from the ostomy pouch. The FP is also regularly communicating with the different specialists that she has seen or is followed by. These include a nephrologist seen every 2 months (who has the most contact with the FP), a urologist (lithotripsy for kidney stones) and a general surgeon (lumpectomy). Appointments with the nephrologist take place at a kidney care clinic where the participant also sees a social worker, usually during the same visit. Home care is provided through the Community Care Access Centre (CCAC), with the participant receiving regular visits from a dietitian (weight gain being the primary objective) and one visit so far from a physiotherapist (to regain muscle strength). The participant has frequented urgent care clinics and the emergency department (ED) in the past when her FP was not available, but generally can call and speak with the FP directly and be seen as early as that same afternoon. The participant has strong connections in the health care community which allowed her to seek out her current FP, after receiving what she perceived as very sub-optimal care from her previous FP. In addition to a strong ability to self-advocate (especially in instances where she perceives care received to be lacking) which she owes partly to her background as a nursing instructor, she has a vast network of supportive family and friends.


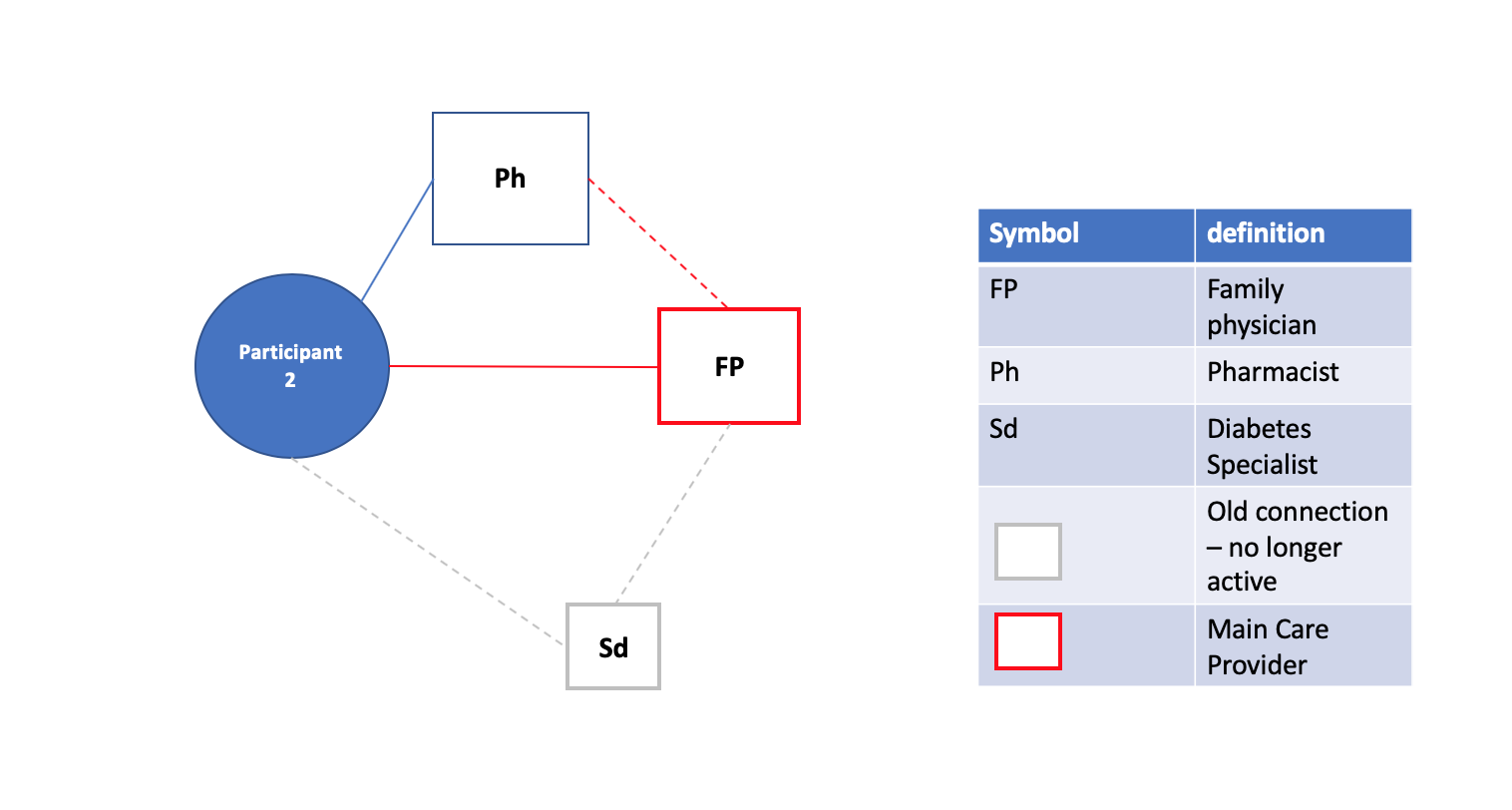


**Participant 2:** 74-year-old male with diabetes and a long history of poor glycemic control. Additionally, he has a multi-year history of peripheral edema in the legs and feet. He was admitted after repeated falls in the home. The participant has been followed by his FP for 17 years. The participant visited them around once per month prior to hospitalization, mostly for management of hi peripheral edema and diabetes. The FP has worked with the participant in the past to manage the swelling by trying and adjusting different medications. Although there were periods of improvement, the edema has always persisted and continued to cause issues with balance and pain. The FP had also referred the patient to a diabetes clinic in the past (during periods of poor blood sugar control), but the specialist who followed the participant has since retired, and management of this issue has fallen back onto the FP. The participant relies heavily on his pharmacist, whom he has a personal relationship with. The participant communicates with him directly when concerns or questions surrounding prescribed medications (and their interactions) arise. The pharmacist does not overstep their duties of care but has on more than one occasion told the participant to go to the hospital, on account of the gravity of the issues the FP seemed content with managing with pharmacological adjustments. The patient does not receive any care from allied health professionals. He is concerned about his ability to properly manage his health problems in the community with his current network of care following discharge.  The patient has some degree of isolation, living far from any potential family supports. His wife is a new Canadian that does not speak English well and who works a demanding job. These factors make it difficult for her to help the participant manage his health care.


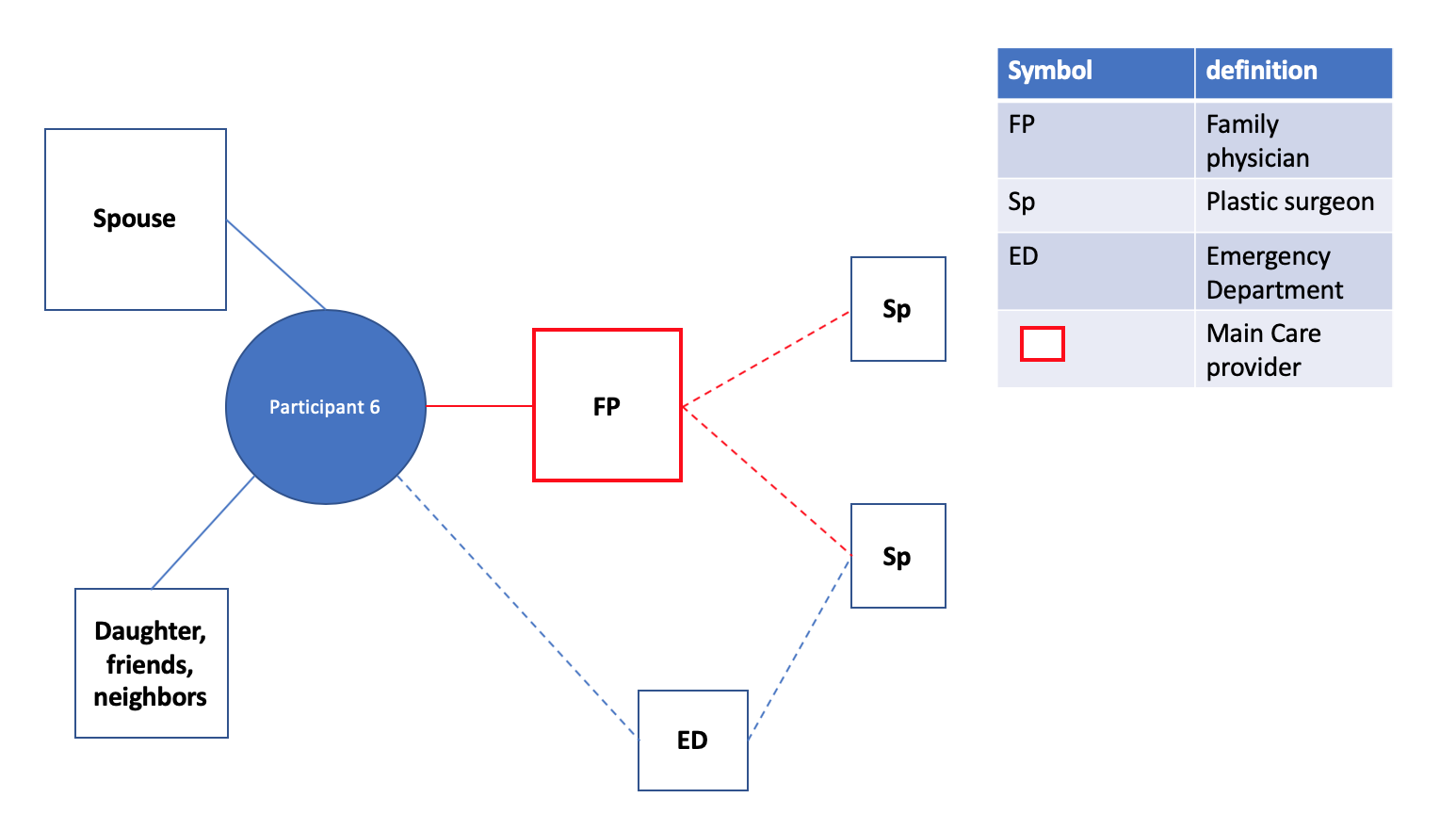


**Participant 6:** 85-year-old man with an extensive past medical history, including a hemorrhagic stroke suffered in 2014, melanoma skin cancer, gastroesophageal reflux disease, diverticulosis and prostate cancer. He is followed by his FP, who he has been rostered with for 10 years. The participant and his wife found a FP when they moved to London, Ontario 20 years ago, but years later were connected with their current FP by their neighbor (circumstances for the change could not be recalled). The participant actively suffers from skin cancer and has already undergone two separate excision procedures. The first was done by his FP, who was not content with the results (due to scarring) and had told the participant they would not do any similar future procedures themselves. The following excision was done by a plastic surgeon who was referred to the participant following a visit to the ED, when the growth had become quite large. The participant was hospitalized shortly after this surgery due to a minor heart attack suffered while visiting his FP. The FP is seen approximately once per month and is the main care provider for this participant. In addition to other responsibilities, they manage the participant’s long list of medications and prioritize health teaching on lifestyle and dietary modifications. The participant has since had another consultation with a different plastic surgeon for another cancerous growth of the skin, this time referred to him by his FP. The participant and his wife note the very long wait times for these appointments and for the excision surgeries as being difficult (they would be willing to pay to have these done faster), but are otherwise very happy with the participant’s arrangement and network of healthcare providers. Following the participant’s stroke in 2014, rehabilitation and home care was provided through CCAC for a 1-year period. The participant’s daughter also provided much needed in-home support during this period. The participant and his wife also have a strong network of friends and neighbors in their lives they can turn to for help. These supports are especially important for the participant’s wife considering her husband was left moderately cognitively and physically impaired by the stroke in 2014. He has no active follow-up for other issues in his past medical history.


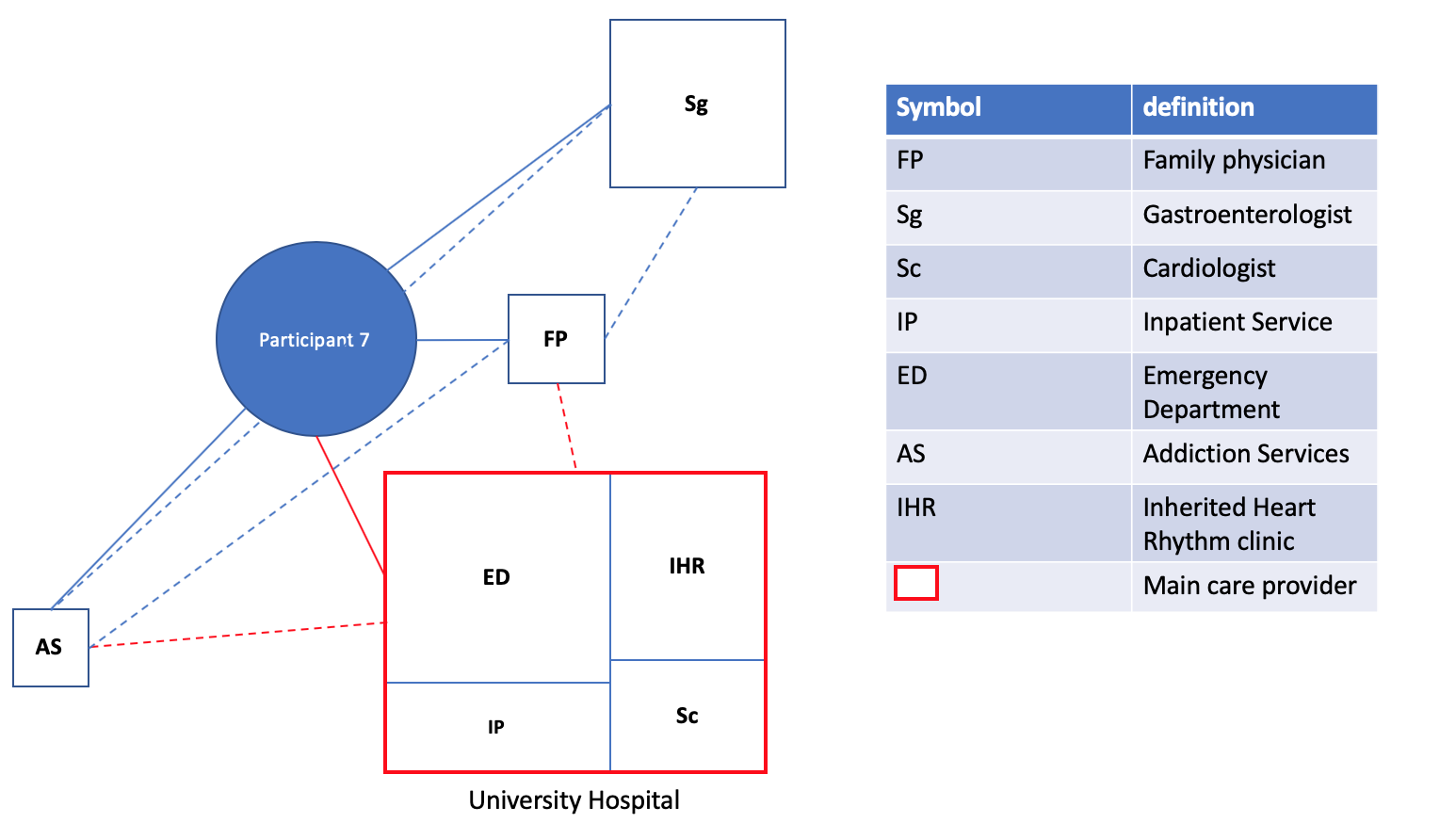


**Participant 7:** 42-year-old male whose active chronic health issues include an inherited heart condition known as Brugada syndrome, chronic pancreatitis, and alcohol use disorder. He was admitted due symptoms related to alcohol withdrawal. The participant has a regular FP at a local clinic, but relies heavily on London, Ontario’s University Hospital. He frequently presents to the ED (sometimes as often as 2-3 times per month), especially in the last year and a half. These admissions often relate to falls and other incidents instigated by either the Brugada syndrome or alcohol use. The patient has been admitted to the internal medicine service at University Hospital before. He had a heart attack in December 2017, which is when he was diagnosed with Brugada syndrome. He had just moved back to London at the time. He now sees a cardiologist infrequently at the hospital (around once per year) and is followed by the Inherited Heart Rhythm Clinic via an implantable cardiac monitor. He makes appointments with the clinic around every 6 months (where he may or may not see his cardiologist), and calls whenever he has a concern or would like to know if a recent fall is related to his heart condition. He is also followed by a gastroenterologist, who he sees approximately every 3 months. He had an acute pancreatitis episode in 2011, and notes he was later told he’d transitioned to chronic pancreatitis by another physician while living outside of London. His gastroenterologist at St. Joseph’s Hospital in London has performed various diagnostic tests including biopsies of the pancreas, and now focuses on optimizing enzyme replacement therapy for the participant. The participant is on a long list of medications for these and other conditions. He notes that his FP sees him often but rushes him out and tells him to defer to his specialists for the health issues which are of most concern to him. He does not feel there is any communication between this physician and his gastroenterologist, and is generally displeased with the lack of the FP’s investment in his overall health care. For his alcohol use disorder, he has been referred by multiple different people to the same addiction service in London. He has not had success with this service and attributes this to a lack of continuity in case managers assigned to him. He is socially isolated, having no family or friend supports here in London to advocate for him, help him manage his health and navigate his different appointments. He considers his main care provider to be University Hospital; this is the central hub as it relates to his health. Whether he is there for Cardiac Care, the ED or admitted to the internal medicine service, he feels he gets the best care here and that this is where his medical history is best known.


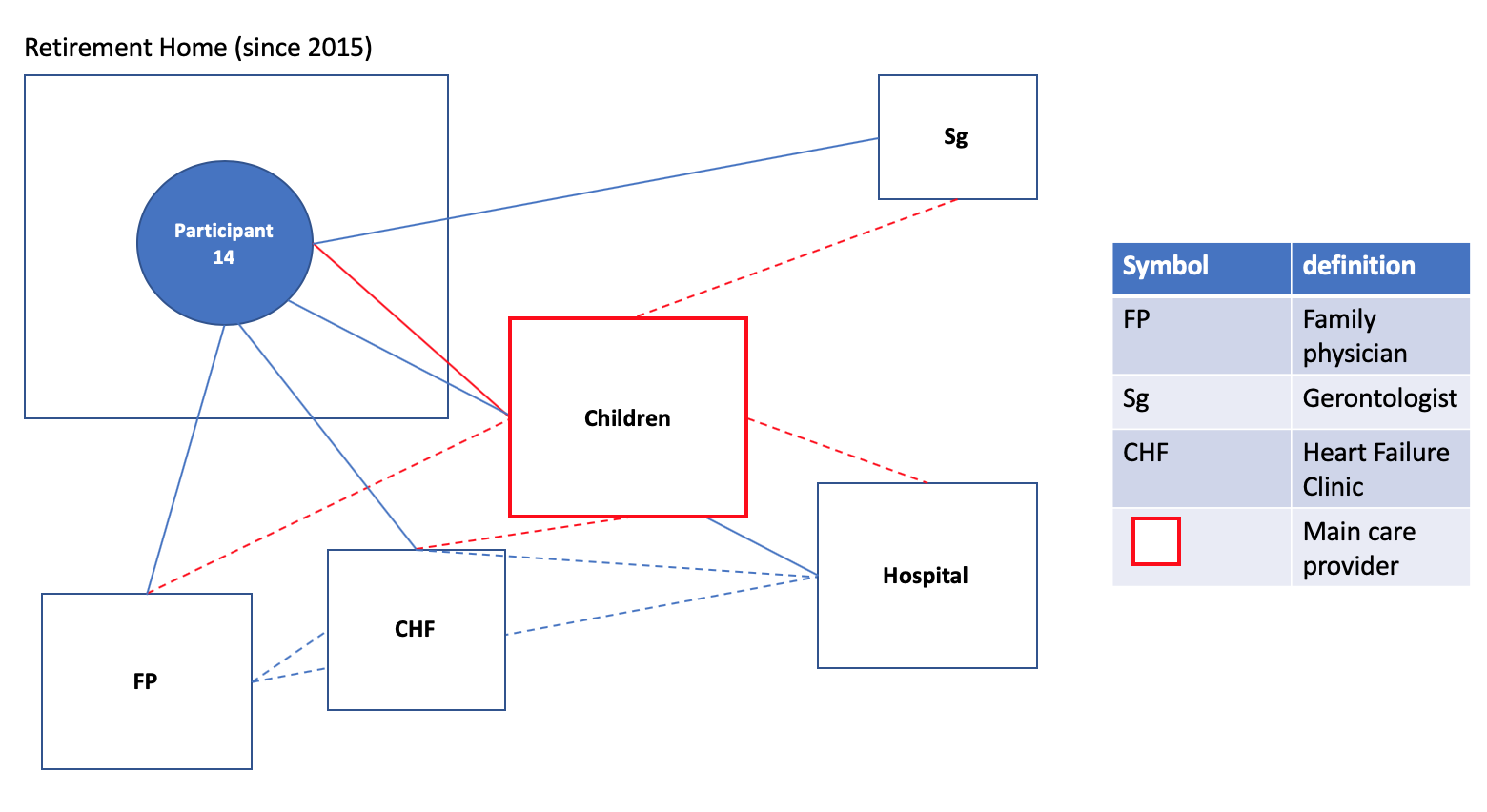


**Participant 12:** 76-year-old male admitted with sepsis. He was not alert or able to participate in the interview, so information was obtained from his daughter. He has COPD and a relatively complex cardiovascular disease history, including a heart attack and two subsequent angioplasties (2004, 2009), two TIAs (2015), an abdominal aortic aneurysm diagnosed in 2017 and more recently a heart failure diagnosis. Following a 2015 admission for sepsis, he was referred to an inpatient rehabilitation program where he was able to regain much of his independence, albeit he has lived in a retirement home with significant support since. He has been with his FP (who he sees at least 6 times per year) for 15 years, and his daughter is quite satisfied with the care he has gotten over the years from this physician. Last November (2018), his daughter had brought him to the ED at University Hospital in London, Ontario due to complaints of chest pain. It was determined he had angina, but the cardiologist he saw during this hospital visit also referred him to the heart failure clinic at St. Joseph’s Hospital in London. He then started visiting the heart failure clinic approximately once per month, where the goal was medical management of his heart condition. His daughter would ensure an appointment with his FP was made shortly after these visits to go over what had been said, results from any tests and medication adjustments. The participant was also followed by a cardiologist up until 2015, but this association somehow dissipated. This is unfortunate, because the participant’s daughter truly believes her father’s heart problems are not amenable to medical management, despite what they have been told at the heart failure clinic and during his last two hospital admissions. Without an active cardiologist to turn to, the participant’s daughter feels like it is very difficult to advocate for the participant’s need to be investigated in the catheterization lab. The participant’s COPD is a relatively recent diagnosis from a few years past. It has been well managed by the FP, and involvement of a respirologist has not been required. The participant’s daughter was upset regarding an abdominal aortic aneurysm diagnosis made during the participant’s 2017 admission which was not communicated to her, the participant, or the FP. She feels that generally, she is the only one who knows what happens when her father is hospitalized, and while she is satisfied with the FP’s involvement, feels that there is only so much they can do. She mentions that despite stressing that the FP be electronically notified of everything that happens, this is not always the case, and she finds herself having to advocate very strongly to ensure there is coordination of care happening upon discharge. The participant also started seeing a gerontologist in 2015, following a decline of his cognitive abilities. His daughter and this physician work together and communicate to ensure the participant is well looked after and properly navigating a rather complex system of different providers. They are looking at a new retirement home for the participant which would offer much more support and be in close proximity to the participant’s FP.


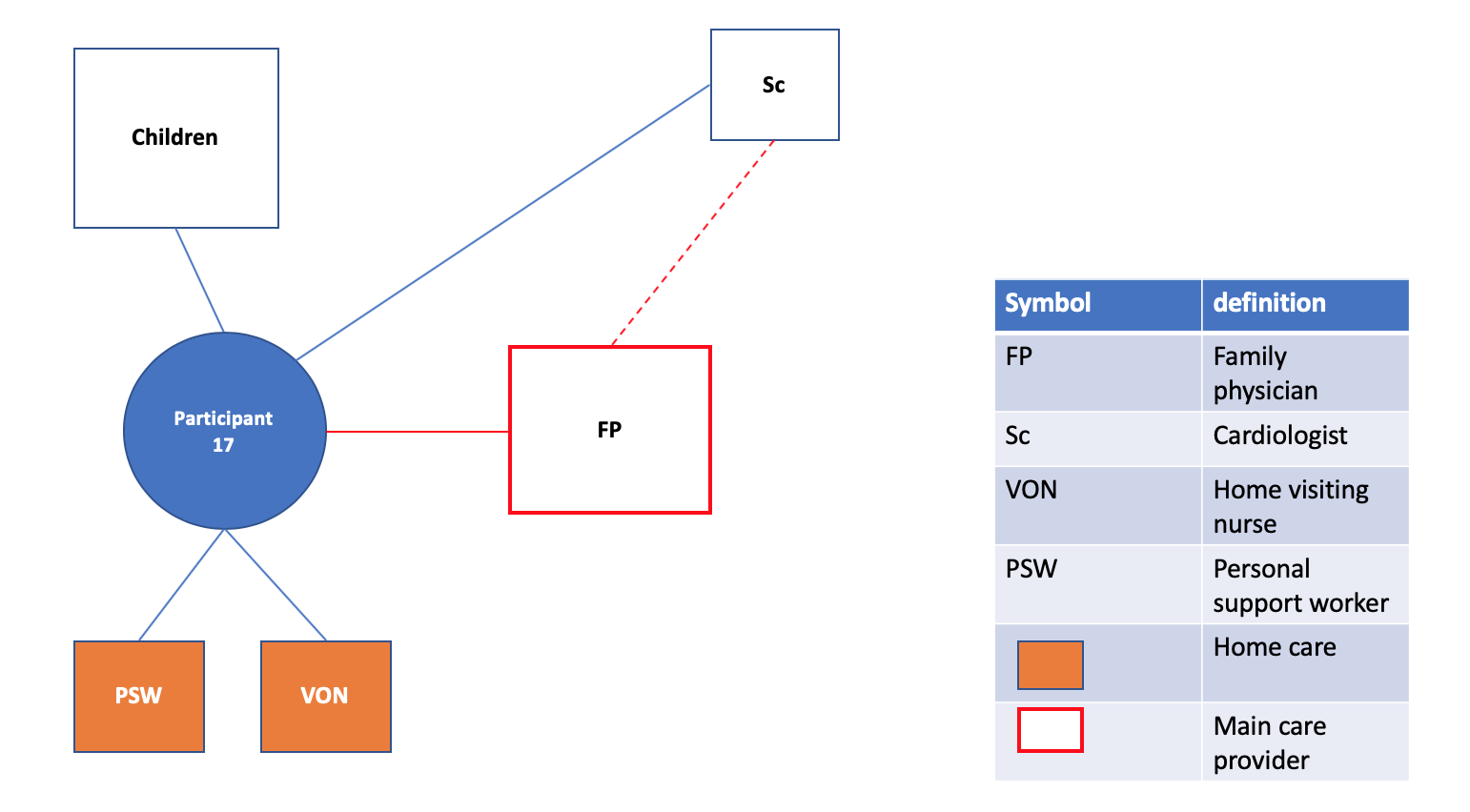


**Participant 17:** 89-year-old female with a history of atrial fibrillation, hypertension, congestive heart failure, chronic back pain, chronic cough, and a papillary cystadenoma resected in 2017. She was admitted with vertigo and functional decline. The participant had a pacemaker implanted during this admission, 4 days prior to her interview. She lives in the community with significant home care (home care nurse 1-3 times per week, and personal support worker three times daily) and support from family members (4 involved children, one of whom lives with the participant as an active caregiver). Her family is at the juncture where they are considering assisted living options due to her ongoing functional decline and the increased burden of her care. The participant has been seeing her FP for the last 7 years. They are actively involved in the management of her hypertension, heart failure, chronic back pain and chronic cough secondary to asthma and reflux. Her FP enrolled her in a home telemonitoring program earlier this year which has now concluded, where the participant would report her vitals electronically every day for 6 months. The daughter also notes that this physician provides education materials to the family, so they know how to react during an exacerbation of the participant’s heart failure. However, there have been 4-5 hospitalizations over the last 2-3 years, and the participant has fallen in the home more than once despite living with one of her children (an active caregiver). A Cardiology referral was made following a hospitalization last July, where congestive heart failure was diagnosed. There have only been two appointments with this specialist however, with another scheduled in the near future.
